# Identification and verification of immune-related genes for diagnosing the progression of atherosclerosis and metabolic syndrome

**DOI:** 10.1186/s12872-024-04026-3

**Published:** 2024-08-02

**Authors:** Qian Xie, Xuehe Zhang, Fen Liu, Junyi Luo, Chang Liu, Zhiyang Zhang, Yining Yang, Xiaomei Li

**Affiliations:** 1 Department of Cardiology, First Affiliated Hospital of Xinjiang Medical University, 137 Liyushan South Road, Urumqi, Tel, 830054 People’s Republic of China; 2 State Key Laboratory of Pathogenesis, Prevention and Treatment of High Incidence Diseases in Central Asia, Clinical Medical Research Institute, the First Affiliated Hospital of Xinjiang Medical University, Urumqi, People’s Republic of China; 3 Department of Cardiology, People’s Hospital of Xinjiang Uyghur Autonomous Region, Urumqi, China; 4 Xinjiang Key Laboratory of Cardiovascular Homeostasis and Regeneration Research, Urumqi, China; 5 State Key Laboratory of Pathogenesis, Prevention and Treatment of High Incidence Diseases in Central Asia, Xinjiang Medical University, Urumqi, China; 6 Key Laboratory of Cardiovascular Disease Research, First Affiliated Hospital of Xinjiang Medical University, Urumqi, China

**Keywords:** Atherosclerosis, Metabolic syndrome, Immune infiltration

## Abstract

**Background:**

Atherosclerosis and metabolic syndrome are the main causes of cardiovascular events, but their underlying mechanisms are not clear. In this study, we focused on identifying genes associated with diagnostic biomarkers and effective therapeutic targets associated with these two diseases.

**Methods:**

Transcriptional data sets of atherosclerosis and metabolic syndrome were obtained from GEO database. The differentially expressed genes were analyzed by RStudio software, and the function-rich and protein-protein interactions of the common differentially expressed genes were analyzed.Furthermore, the hub gene was screened by Cytoscape software, and the immune infiltration of hub gens was analyzed. Finally, relevant clinical blood samples were collected for qRT-PCR verification of the three most important hub genes.

**Results:**

A total of 1242 differential genes (778 up-regulated genes and 464 down-regulated genes) were screened from GSE28829 data set. A total of 1021 differential genes (492 up-regulated genes and 529 down-regulated genes) were screened from the data set GSE98895. Then 23 up-regulated genes and 11 down-regulated genes were screened by venn diagram. Functional enrichment analysis showed that cytokines and immune activation were involved in the occurrence and development of these two diseases. Through the construction of the Protein–Protein Interaction(PPI) network and Cytoscape software analysis, we finally screened 10 hub genes. The immune infiltration analysis was further improved. The results showed that the infiltration scores of 7 kinds of immune cells in GSE28829 were significantly different among groups (Wilcoxon Test < 0.05), while in GSE98895, the infiltration scores of 4 kinds of immune cells were significantly different between groups (Wilcoxon Test < 0.05). Spearman method was used to analyze the correlation between the expression of 10 key genes and 22 kinds of immune cell infiltration scores in two data sets. The results showed that there were 42 pairs of significant correlations between 10 genes and 22 kinds of immune cells in GSE28829 (|Cor| > 0.3 & *P* < 0.05). There were 41 pairs of significant correlations between 10 genes and 22 kinds of immune cells in GSE98895 (|Cor| > 0.3 & *P* < 0.05). Finally, our results identified 10 small molecules with the highest absolute enrichment value, and the three most significant key genes (*CX3CR1*,* TLR5*,* IL32*) were further verified in the data expression matrix and clinical blood samples.

**Conclusion:**

We have established a co-expression network between atherosclerotic progression and metabolic syndrome, and identified key genes between the two diseases. Through the method of bioinformatics, we finally obtained 10 hub genes in As and MS, and selected 3 of the most significant genes (*CX3CR1*,* IL32*,* TLR5*) for blood PCR verification. This may be helpful to provide new research ideas for the diagnosis and treatment of AS complicated with MS.

## Introduction

Atherosclerosis (AS) is a systemic disease with limited manifestations, and it is also one of the main causes of cardiovascular disease (CVDs) and one of the leading causes of death worldwide [1]. With the occurrence and development of atherosclerosis, it will lead to more serious cardiovascular adverse events, such as acute myocardial infarction and acute stroke, and even fatal sudden cardiac death [2, 3]. Therefore, the diagnosis and timely treatment of high-risk plaques in patients is of great significance to reduce cardiovascular events [4].

Metabolic syndrome (MS) is a chronic non-infectious syndrome characterized by a series of vascular risk factors, including insulin resistance, hypertension, abdominal obesity, impaired glucose metabolism and dyslipidemia.These risk factors are caused by pro-inflammatory state, oxidative stress, hemodynamic dysfunction and ischemia [5]. At the same time, MS plays an important role in the process of atherosclerosis, and clustering of related risk factors may increase the risk of atherosclerotic injury. There is a correlation between MS components and the progression of atherosclerosis, and atherosclerosis is the main cause of cardiovascular death [6]. Previous studies have identified and verified the coexpression of genes between AS and Diabetes [7]. However, for Metabolic Syndrome, which is more complex than Diabetes, there is less systematic relationship between AS and MS at the genetic level. So identifying new diagnostic markers and related treatment targets is of particular significance for the diagnosis and new treatment of patients with atherosclerosis and metabolic syndrome.

To further explore the potential interaction between AS and MS, we have obtained a database (Gene Expression Omnibus database, GEO) containing early and advanced / late atherosclerotic plaques (GSE28829) and a Metabolic Syndrome (GSE98895) related expression profile from the gene expression data sets. After the differential analysis of the samples of AS and MS, the related differentially expressed genes were obtained. At the same time, the AS-related differential genes were intersected with MS-related differential genes, and the common differential genes were identified by Gene Ontology (GO)/Kyoto Encyclopedia of Genes and Genomes (KEGG) enrichment analysis and the Protein–Protein Interaction (PPI) interaction network. The key genes were obtained by Cytoscape software, and the clinical related blood samples were collected for verification.

## Materials and methods

###  Data acquisition and download

**Fig. 1:**
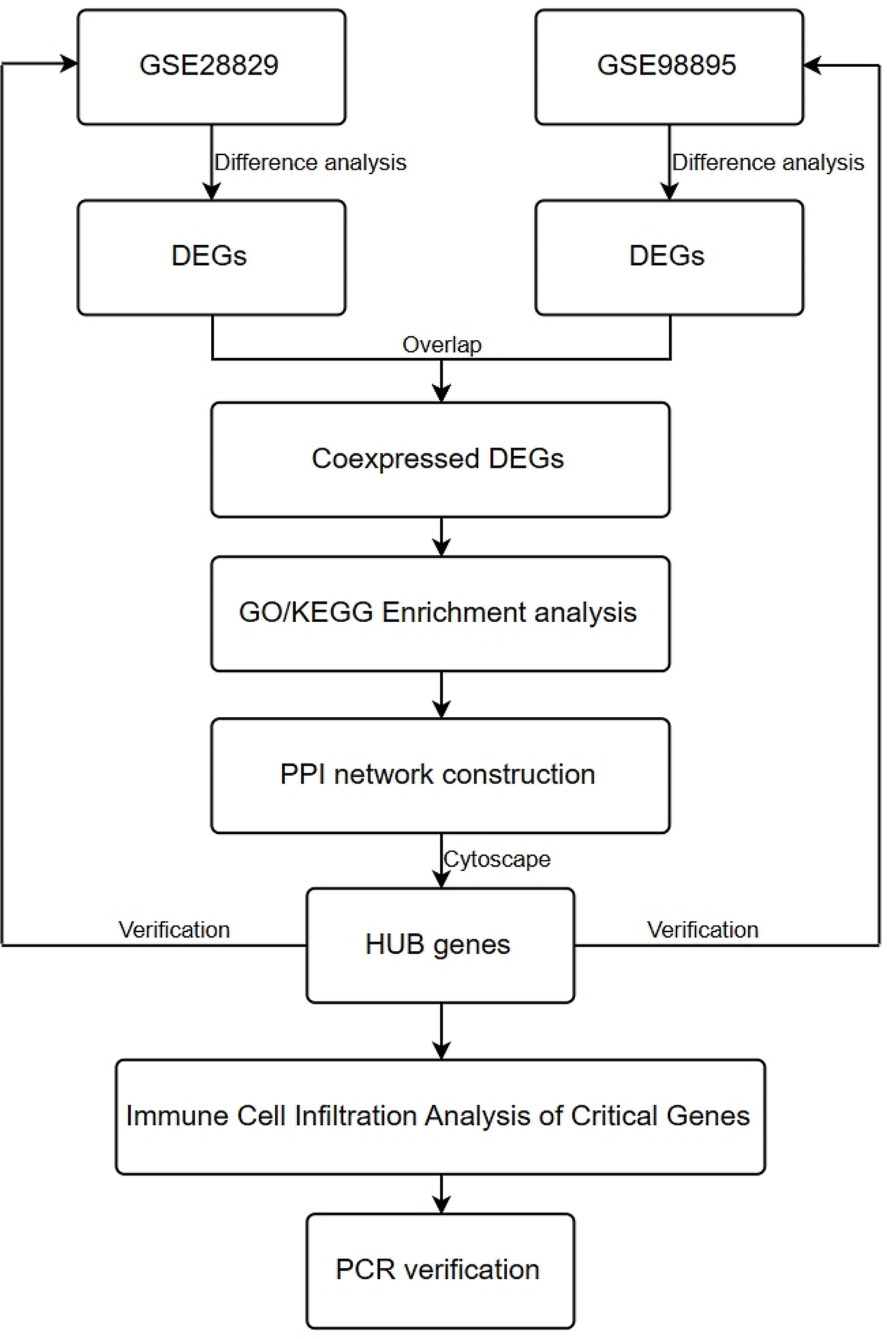
Workflow of this study

The study flowchart is shown in Fig. 1. In the methodology of our study, multiple database were procured from the Gene Expression Omnibus (GEO, https://www.ncbi.nlm.nih.gov/geo/) database to carry out comprehensive analyses.The GSE28829 (Last update: Mar 25, 2019) atherosclerosis data set was selected, including 13 early (intimal thickening and intimal xanthoma) and 16 late (thin or thick fibrous cap) carotid plaque samples. And GSE98895 (Last update: Jul 25, 2021) Metabolic Syndrome data set, including 20 metabolic syndrome patients’ peripheral blood monocyte sequencing data and 20 non-metabolic syndrome patients’ data sets were included in the analysis (Table 1).

**Table 1:** Summary of the datasets used in this study

GEO Accession	Platform	Tissue Type	Samples
GSE28829	GPL570	Early and advanced atherosclerotic plaques	13VS16
GSE98895	GPL6947	Peripheral blood mononuclear lymphocytes in healthy people and patients with metabolic syndrome	20VS20

### Differentially expressed gene identification

To achieve a comprehensive and consistent analysis of our multi-dataset genomic study, several strategic methodologies were employed. Initially, addressing potential batch effects was imperative, particularly as they could arise from diverse experimental conditions or unforeseen technical discrepancies. The R software (4.2.1) is used for data processing. GSE28829 and GSE98895 are downloaded from the GEO database through the GEOquery package. Standardize the data again through the normalizeBetweenArrays function of the Limma package to remove the probe corresponding to multiple molecules. We have matched probes and gene symbols via the Bioconductor package [8] with the corresponding annotation document; when the probe corresponding to the same molecule is encountered, only the probe with the largest signal value is retained, and the visualization of the difference result.We Used the Limma package to analyze the difference between the two groups, only the genes with *P* < 0.05 and |logFC|> 0.5 are considered to be meaningful differentially expressed genes (DEGs). The results of difference analysis are visualized by heatmap, and the significantly expressed molecules are visualized in the form of volcano plot.

###  Enrichment analysis of common DEGs

Overlap the common up-and down-regulated significant DEGs shared by GSE28829 and GSE98895 datasets, and the results are shown using the venn diagram.The overlapping genes were enriched and analyzed by Gene Ontology (GO) [9] and Kyoto Encyclopedia of Genes and Genomes (KEGG) [10] using R software clusterProfiler package.

###  Construction of PPI interaction network and screening of HUB genes

The Protein–Protein Interaction (PPI) network is a mathematical representation of the physical relationship of candidate genes at the protein level, which is mainly used to further understand the pathogenesis of diseases and drug-related therapeutic targets [11, 12]. PPI network was based on STRING database (https://cn.string-db.org), and its minimum interaction score (comprehensive score > 0.15). Interactive information was downloaded and used Cytoscape software (version 3.9.2) to visualize.The plug-in MCODE (version 2.0.2) was used to find the central sub-network of the protein-protein interaction network, and the node gene contained in the central sub-network with the highest score was selected as the hub genes.

###  Immune infiltration and immune correlation analysis of HUB genes

Based on the expression profile data sets of GSE28829 and GSE98895, the relative scores of immune infiltration of 22 kinds of immune cells in all samples of the two data sets were evaluated by Cibersort method in R packet IOBR [13]. Then, combined with the sample grouping information of the data set, the differences of immune infiltration scores between Early atherosclerotic plaques group and Advanced atherosclerotic plaques group in GSE28829 and between MS group and Control group in GSE98895 were compared and analyzed. Based on the results of hub genes identification and immune infiltration analysis, Spearman method was used to analyze the correlation between the expression of 10 key genes and 22 kinds of immune cell infiltration scores in two data sets.

###  Verification of HUB genes expression

All identified hub genes were further verified by GSE28829 and GSE98895 to avoid false positive rates. Wilcox test was used to compare the early group and the late group, the MS group and the non-MS group. *P* < 0.05 means that the difference between groups is statistically significant.

###  Clinical specimen collection and qRT-PCR analysis

In this study, consecutive patients who were hospitalized in the Heart Center of the first affiliated Hospital of Xinjiang Medical University from January 2023 to September 2023, all the participants were uniformly informed of the purpose of the study by the doctor before admission, and all the patients signed the informed consent form before participating in this study. Through the further integration of the diagnosis, examination and other information of the patients, the people who accord with the diagnosis of metabolic syndrome and improve the carotid artery ultrasound examination are selected and included in this study. Metabolic syndrome is defined as any three or more of the following: Waistline > 102 cm in men and > 88 cm in women; Blood Pressure > 130/85 mmHg or taking medication; Fasting Blood Glucose (FPG) ≥ 110 mg/dL or taking medication; Triglyceride (TG) ≥ 150 mg / dL; HDL-C < 40 mg / dL in males and < 50 mg / dL in females [14]. Exclusion criteria: acute coronary syndrome, moderate / severe valvular disease, acute decompensation and / or severe heart failure, acute / chronic inflammatory infectious diseases, inflammatory / autoimmune diseases, severe liver and kidney diseases, hematological diseases and malignant tumors, and patients exposed to alcohol or other drugs. This study was approved by the Ethics Committee of the first affiliated Hospital of Xinjiang Medical University (approval number: 20220308-105). RNA was extracted from peripheral blood using Trizol reagent (Invitgen, US) and cDNA was synthesized using reverse transcription kit (Applied Biological Systems). Real-time quantitative polymerase chain reaction was carried out on BioRad CFX96 using KAPA SYBR Green FAST BioRad Cycler Kapa kit (PeqLab). The expression of target genes was detected by 2 − ΔΔ-β-actin Ct method. Primer sequence: *CX3CR1*: 5’-CTGCCTCTTAGACTTCTG-3’(forward), 5’-GGCTATCACTCTGTAGAC-3’(reverse). *IL-32*: 5’-CGACTTCAGAGTGCATGTT-3’(forward),5’-TGTTGCCTCTGAGTCGTAATTC-3’(reverse).*TLR5*: 5-’TCTCCAGGATGTTGGCTGGTTTCT-3’(forward), 5’-AAAGTTCTTGGCTCACTAGGGCGA-3’(reverse).

###  Statistical analysis

Rstudio (4.2.1) software was used for drawing and statistical analysis. The continuity variable is expressed by mean ± standard deviation (SD) or median (P25 and P75). Classification variables are expressed by the number of cases and percentage (%). *P* < 0.05 indicated statistical significance.

## Results

### Co-Expression differential genes of AS and MS

**Fig. 2:**
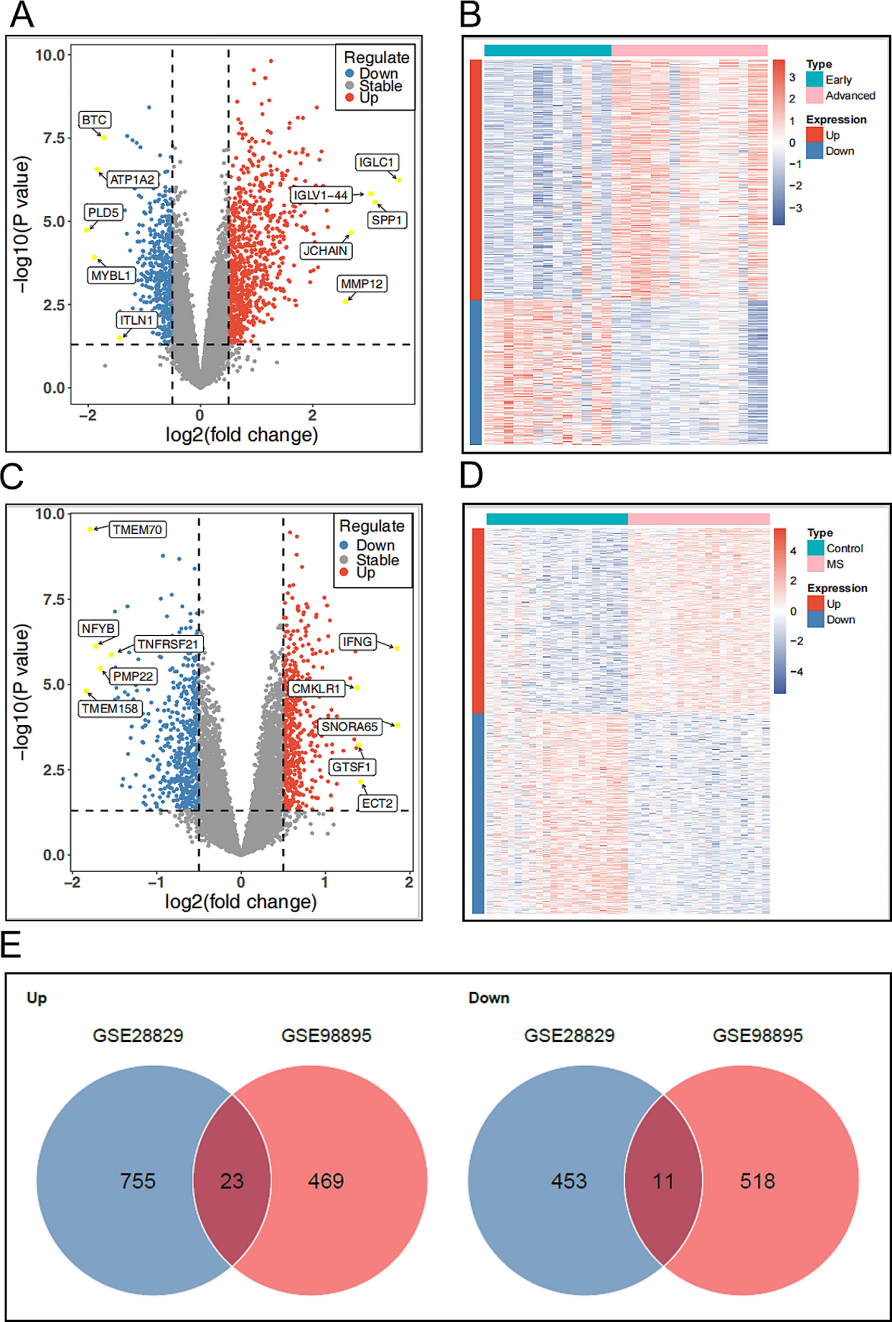
Identification of DEGs and screening of common differential genes.(**A**)Volcano plot of DEGs of AS. (**B**) Heatmap of DEGs of AS. (**C**)Volcano plot of DEGs of MS. (**D**) Heatmap of DEGs of MS. (**E**) Venn diagram of co-up-regulated and down-regulated genes of AS and MS

To identify genes co-expressed in AS and MS, the microarray data came from two data sets: GSE28829 (13 early and 16 late plaque samples) and GSE98895 (20 metabolic syndrome and 20 non-metabolic syndrome patients) for training sets (Table 1). After the normalization and logarithmic processing of the data, the probes without annotated information are removed, and the R software was used to calculate the average value in the presence of repeated expression data. The genes with screening criteria of *P* < 0.05 and | logFC | > 0.5 were identified as DEGs. A total of 1242 differential genes (778 up-regulated genes and 464 down-regulated genes) were screened from GSE28829 data set (Fig. 2A-B). A total of 1021 differential genes (492 up-regulated genes and 529 down-regulated genes) were screened from the data set GSE98895 (Fig. 2C- D). The differential expression results are visualized by heatmap and volcano plot respectively. Then, we extracted the co-expressed genes between the two data sets, and screened 23 up-regulated and 11 down-regulated genes as potential crosstalk genes by Venn diagram, indicating that there may be a common pathogenesis between AS and MS (Fig. 2E).

### Functional enrichment analysis of Co-Expression differential genes

**Fig. 3:**
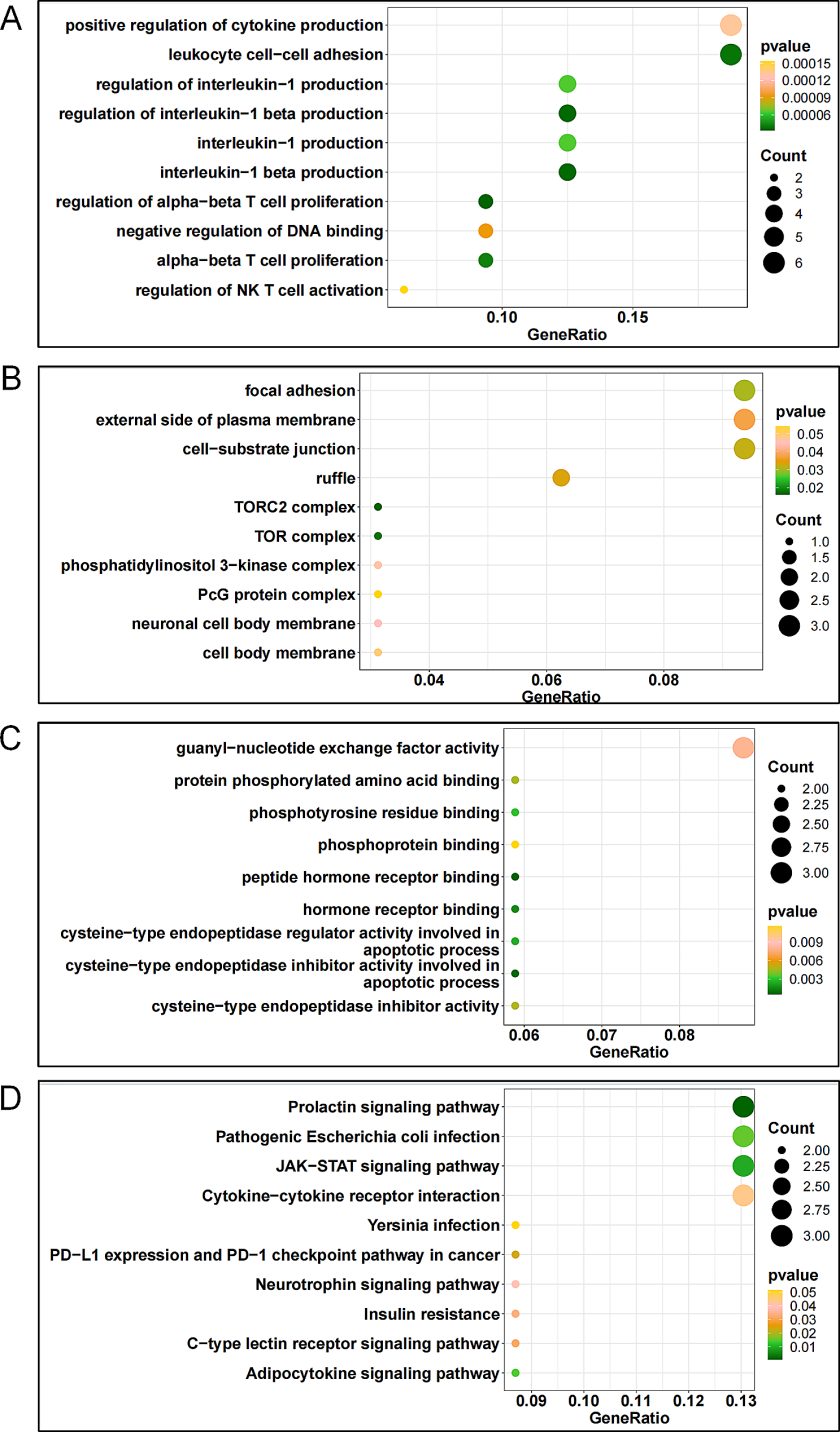
Functional enrichment Analysis of Common differential genes between AS and MS. (**A**) BP (biological process) enrichment of differential genes. (**B**) CC(cellular component) enrichment of differential genes. (**C**)MF (molecular function) enrichment of differential genes.(**D**) KEGG enrichment of differential genes

In order to further analyze the biological functions and pathways of common differential genes, we used R software clusterProfiler package to analyze the enrichment of GO and KEGG.In GO enrichment, overlapping differential genes were enriched into three types of functions: BP (biological process), MF (molecular function) and CC (cellular component), with a total of 1354 items (MF: 123; CC: 62; BP: 1169). The results indicate that BP mainly in positive regulation of cytokine production, leukocyte cell − cell adhesion, regulation of interleukin − 1 production, regulation of interleukin − 1 beta production, interleukin − 1 production, interleukin − 1 beta production. CC is primarily in focal adhesion, external side of plasma membrane, cell − substrate junction, ruffle. MF is primarily in guanyl − nucleotide exchange factor activity (Fig. 3A-B-C).

In KEGG enrichment, 75 pathways were enriched by overlapping differential genes.The results show the top 10 pathways in p.adjust, including Prolactin signaling pathway, *Pathogenic Escherichia coli* infection, JAK − STAT signaling pathway, Cytokine − cytokine receptor interaction, *Yersinia* infection, PD − L1 expression and PD − 1 checkpoint pathway in cancer, Neurotrophin signaling pathway, Insulin resistance, C − type lectin receptor signaling pathway, Adipocytokine signaling pathway (Fig. 3D).

### Construction of PPI interaction network and screening of HUB Genes

**Fig. 4:**
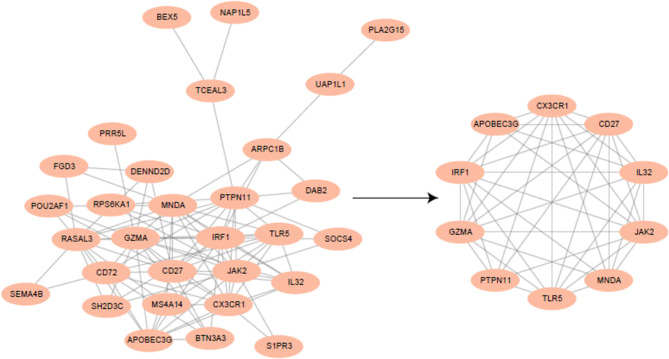
Construction of PPI Network and screening of hub Genes

In order to further clarify the interaction of differential genes between AS and MS, based on 34 differentially expressed genes, we used STRING database prediction (Confidence > 0.15), protein interaction network and Cytoscape for visualization.Among the 34 differential genes, 30 genes could predict 98 interactions. Then, we used the Cytoscape software MCODE algorithm to find the central sub-network of the protein-protein interaction network, and select the node genes contained in the central sub-network with the highest score as the core genes.The results showed that 10 genes including *APOBEC3G*,* CD27*,* CX3CR1*,* GZMA*,* IL32*,* IRF1*,* JAK2*,* MNDA*,* PTPN11* and *TLR5* were identified as key genes (Fig. 4).

### Immune infiltration and immune correlation analysis of HUB genes

**Fig. 5:**
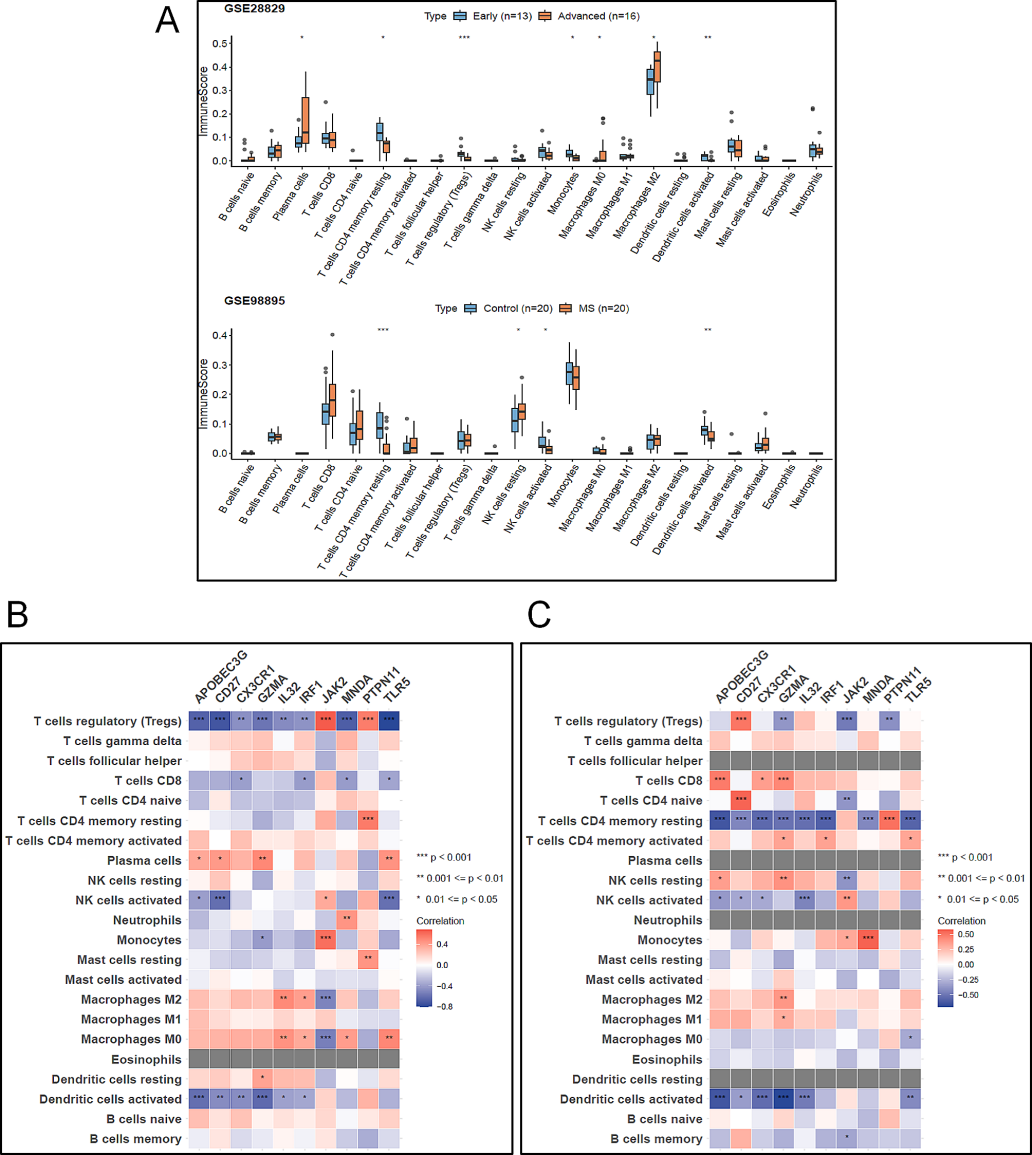
Pertinence of the critical genes with immune cells. (**A**) Boxplots of 22 infiltrating immune cells in GSE43292 and GSE25724 data sets. (**B** and **C**) Correlations between immune cells and ten critical genes

Immunity plays a key role in atherosclerosis and metabolic syndrome. In order to clarify the interaction between AS and MS, we compared and analyzed the difference of immune infiltration score between Early group and Advanced group in GSE28829 and between MS group and Control group in GSE98895. The results showed that there were significant differences in the infiltration scores of 7 kinds of immunocytes in GSE28829, including Plasma cells, T cells CD4 memory resting, T cells regulatory (Tregs), Monocytes, Macrophages M0, Macrophages M2 and Dendritic cells activated between groups (Wilcoxon Test < 0.05). In GSE98895, the infiltration scores of four kinds of immunocytes, including T cells CD4 memory resting, NK cells resting, NK cells activated and Dendritic cells activated, were significantly different among groups (Wilcoxon Test < 0.05) (Fig. 5A).

Based on the results of key gene identification and immune infiltration analysis, Spearman method was used to analyze the correlation between the expression of 10 key genes and 22 kinds of immune cell infiltration scores in two data sets. The results showed that there were 42 pairs of significant correlations between 10 genes and 22 kinds of immune cells in GSE28829 (|Cor| > 0.3 & *P* < 0.05), and there was a significant correlation between the immune infiltration score of T cells regulatory (Tregs) and the expression of 10 genes (Fig. 5B). There were 41 pairs of significant correlations between 10 genes and 22 kinds of immune cells in GSE98895 (| Cor | > 0.3 & *P* < 0.05). Among them, there was a significant correlation between the immune infiltration score of T cells CD4 memory resting and the expression of 9 genes (Fig. 5C).

###  Verification of HUB genes

**Fig. 6:**
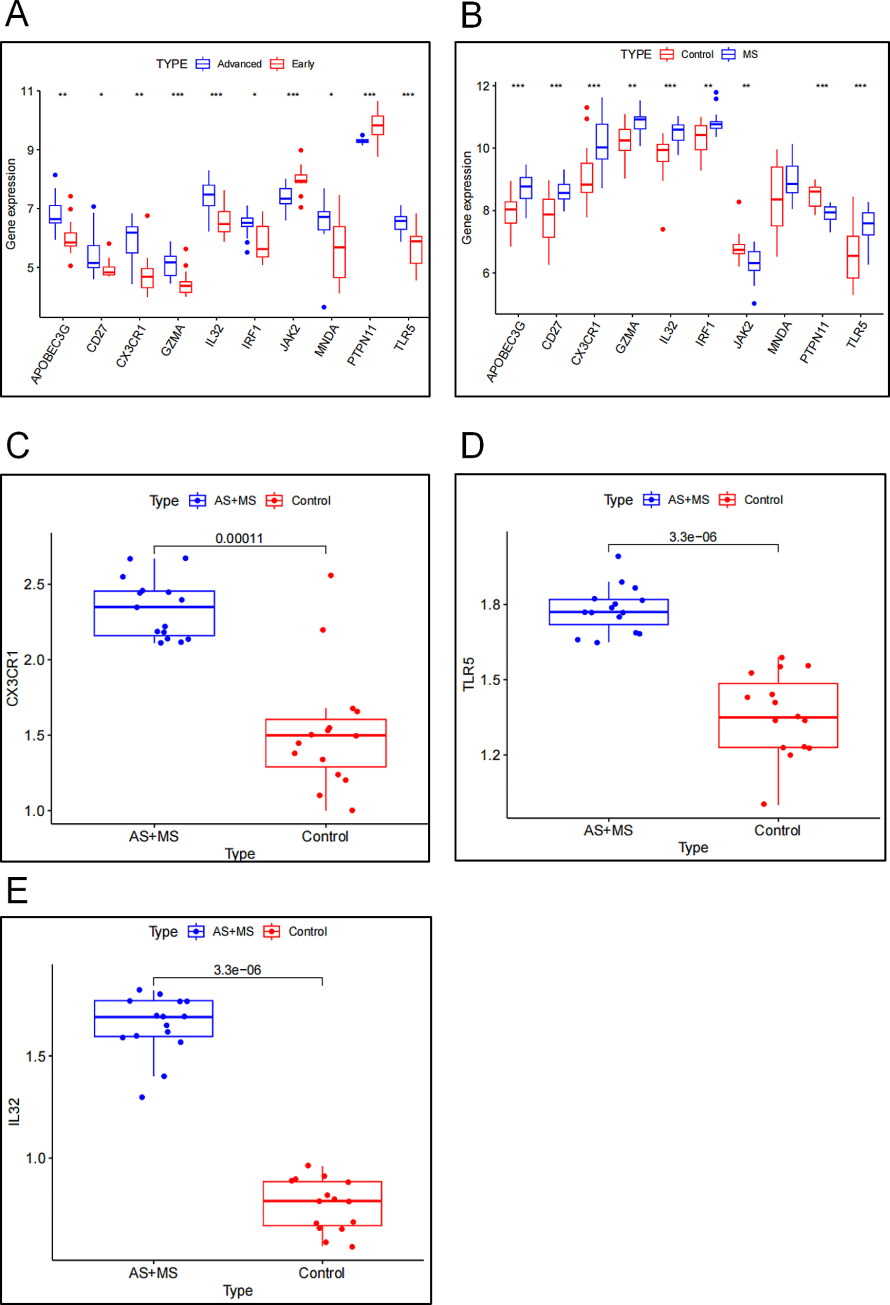
Verification of hub genes. (**A**) Verification of hub Gene in AS dataset. (**B**) Verification of hub Gene in MS data set.(**C**-**E**) PCR results of *CX3CR1*,* TLR5* and *IL32* in Human Blood samples

In order to further verify the reliability of the selected 10 hub genes, we have chosed to further verify the expression of 10 hub genes in the GSE28829 and GSE98895 data expression matrix.Results as shown in the Fig. 6, in the GSE28829 data set, 10 hub genes *APOBEC3G*,* CD27*,* CX3CR1*,* GZMA*,* IL32*,* IRF1*,* MNDA* and *TLR5* were up-regulated and statistically significant in advanced / advanced atherosclerotic tissues, while *JAK2* and *PTPN11* were down-regulated in advanced atherosclerotic tissues (Fig. 6A). In the GSE98895 data set, *APOBEC3G*,* CD27*,* CX3CR1*,* GZMA*,* IL32*,* IRF1* and *TLR5* were up-regulated in MS patients, while *JAK2* and *PTPN11* were down-regulated in MS patients, which were consistent with the results of AS (Fig. 6B). In order to further clarify the significance of hub gens in patients, A total of 60 patients were randomly selected for this study. According to the diagnosis of metabolic syndrome complicated with carotid atherosclerotic plaque, the patients were divided into control group and case group. The baseline characteristics of all participants are shown in Table 2. There was no significant difference in total cholesterol, low density lipoprotein, diastolic blood pressure and serum creatinine between the two groups. The patients in the case group were older and had more males. BMI, triglyceride, high density lipoprotein, systolic blood pressure, blood glucose, prevalence of hypertension and prevalence of diabetes were higher than those in the control group. Next, we selected the first three up-regulated genes of hub genes (*CX3CR1*,* IL32*,* TLR5*), and further detected their gene expression in peripheral blood by PCR. The results showed that the expression of these three genes increased significantly in the case group (Fig. 6C-D-E).

**Table 2:** Baseline characteristics of patients

	Case(*n* = 30)	Control(*n* = 30)	*P* Value
Age (years)	60.30 ± 12.46	52.23 ± 8.79	<0.05
Gender (male)	19(63.3%)	9(26.7%)	<0.05
BMI (kg/m2)	27.31(26.43,28.82)	21.70(19.95,22.97)	<0.001
Total cholesterol (mmol/L)	3.94(3.16, 4.35)	4.48(3.52, 4.66)	0.19
Triglycerides (mmol/L)	2.21(1.64, 2.78)	0.99(0.65,1.52)	<0.001
LDL cholesterol (mmol/L)	2.36(1.99, 3.41)	2.53(1.93, 2.88)	0.593
HDL cholesterol (mmol/L)	0.84(0.71, 0.96)	1.23(1.10, 1.42)	<0.001
SBP(mmHg)	135.5(122.75, 145.25)	116.15(109.75, 123.25)	<0.001
DBP(mmHg)	79(71.75, 84.25)	75.5(69, 80)	0.131
Blood sugar(mmol/L)	6.13(4.83, 7.09)	4.63(4.23, 5.06)	<0.05
HbA1c (%)	6.10(5.5, 6.96)	5.66(5.5, 5.81)	0.048
Creatinine(µmol/L)	67.90(57.26, 85.11)	65.95(51, 71.03)	0.107
Hypertension (%)	27(90%)	5(16.7%)	<0.001
Diabetes mellitus (%)	12(40%)	1(3.3%)	<0.001

## Discussion

There is a close relationship between atherosclerosis and metabolic syndrome. MS plays an important role in the occurrence and development of AS. MS is a multiple risk factor for atherosclerotic cardiovascular disease, but the specific relationship between MS and AS is not completely clear.In our study, we used GEO database to obtain common differential genes between AS and MS through difference analysis, and used GO/KEGG functional enrichment analysis to explore their possible related biological processes. Then we established a PPI interaction network and identified 10 hub genes using Cytoscape software. Finally, we performed PCR in GEO database and plasma of metabolic syndrome patients with atherosclerosis to further verify the screened hub gene. Finally, we screened 10 hub genes, and there were significant differences among patients with AS complicated with MS.

Atherosclerosis refers to the accumulation of fat and / or fibrous substances in the innermost layer of the artery, namely intima. Over time, atherosclerotic plaques become more fibrous and accumulate calcium minerals. Late atherosclerotic plaques can invade the arterial lumen, hinder blood flow, and lead to tissue ischemia. Atherosclerosis, which does not produce flow-limiting blockage, destroys and causes thrombosis, which blocks the lumen, providing a second pathway to ischemia, usually more acute. Atherosclerotic cardiovascular disease (CVDs) is still the leading cause of vascular disease worldwide. When it affects the circulation of the heart itself, it can cause acute coronary syndrome or chronic diseases, including myocardial infarction, such as stable angina pectoris (chest pain or discomfort caused by insufficient myocardial perfusion). Atherosclerosis causes many ischemic strokes and transient ischemic attacks. It can lead to the formation of aneurysms, including those on the abdominal aorta. When it affects the peripheral artery, it can cause intermittent claudication, ulcers and gangrene, endangering the viability of the limbs [15].

MS is a multiple risk factor associated with metabolic abnormalities [16]. MS is characterized by a series of interrelated risk factors for atherosclerosis, including insulin resistance, hypertension, abdominal obesity, impaired glucose metabolism and dyslipidemia, which share the risk of ASCVD.Having three or more of these ingredients will make it possible for a person to have MS [17]. Detailed understanding of the components of MS is essential for the development of effective prevention strategies and appropriate intervention tools, which can curb its increasing prevalence and limit its complications.Hyperglycemia is considered to be a component of MS. It is described as a steady state with higher-than-normal plasma glucose levels after overnight fasting.The underlying pathophysiological mechanism is the interaction between pancreatic β-cell dysfunction and peripheral and hepatic IR, which leads to abnormal hepatic glucose production [18]. Due to the use of insulin or hypoglycemic drugs, diabetic patients rarely die of hyperglycemia; on the contrary, 75% of diabetic patients die directly from cardiovascular disease [19]. The risk of cardiovascular disease in patients with diabetes is 2–4 times higher than that in the general population [20]. Hypertension is another important component of MS, which exists in up to 1/3 of MS patients.There is evidence that even if there is no T2DM, MS can increase the risk of cardiovascular morbidity and mortality in patients with hypertension [21]. Blood pressure level is closely related to visceral obesity and insulin resistance, which is the main pathophysiological feature of MS. The higher level of systolic blood pressure may reflect the progressive hardening of arterial wall, the change of vascular structure and the development of atherosclerosis [22]. Obesity is a multifactorial chronic disease characterized by fat deposition in new adipocytes and enlargement of existing cells [23]. Obesity is a chronic inflammatory state that produces a variety of cytokines and inflammatory markers that increase the risk of cardiac metabolism and metabolism-related diseases [24, 25]. Obesity can be quantified by body mass index (BMI), which is determined by weight (kg) divided by height squared (m2) (kg/m2). The BMI index is determined by weight (kg) divided by height squared (m2). A better way to define obesity is by the percentage of total body fat [26]. Body fat percentage measurements are rarely used because of inconvenience and cost, so the best way to estimate obesity is to calculate the waist circumference (WC). This is because excessive abdominal fat is closely related to metabolic risk factors.Waist circumference ratio (WHR) is an alternative indicator of central obesity. Compared with BMI [27] and WC [28], WHR is a superior indicator of CVD risk. Studies have shown that each additional unit of BMI increases the risk of cardiovascular disease by 8 per cent [29]. In addition, for every 0.01 unit increase in waist width ratio for both men and women, the risk of cardiovascular events increased by 5% [28]. Therefore, these simple indicators of abdominal obesity should be included in the risk assessment of cardiovascular disease. Weight control through lifestyle changes is considered to be an effective strategy to achieve and maintain a healthy weight.Lipid abnormality is a sign of MS, which is characterized by an increase in plasma triglyceride concentration, a decrease in high density lipoprotein cholesterol (HDL-C) and an increase in low density lipoprotein cholesterol (LDL-C).Dyslipidemia is generally considered to be an independent risk factor for atherosclerosis [30]. Low plasma HDL-C level and hypertriglyceridemia are independently and significantly correlated with myocardial infarction in patients with MS [31]. Therefore, in our study, we found 10 meaningful hub genes between the common differentially expressed genes of AS and MS, and verified by PCR by collecting relevant clinical blood samples. the results showed that there were significant differences in hub genes between patients and non-patients. We focused on the three key genes we verified.

*CX3CR1* is the receptor of *CX3CL1*, which is a G protein coupled receptor (GPCR). It has seven transmembrane (TM7) transmembrane regions. Under the condition of flow in vitro, *CX3CR1* receptor can mediate the tight adhesion of cells to fixed fractalkine. *CX3CR1* exists in many early leukocyte cells, and *CX3CR1-CX3CL1* signal transduction plays different functions in different tissue regions, such as immune response, inflammation, cell adhesion and chemotaxis [32]. *CX3CR1-CX3CL1* signal transduction mediates cell migration function (through similarity). Responsible for recruiting natural killer (NK) cells into inflamed tissue (through similarity).Promote cell survival (through similarity) by mediating macrophages and monocytes to recruit inflamed atherosclerotic plaques as regulators of the inflammatory process that leads to atherosclerosis. *CX3CL1* and *CX3CR1* play a role in many inflammatory diseases. It has been suggested that *CX3CR1* participates in the pathogenesis of these diseases by promoting the migration of monocytes or lymphocytes expressing *CX3CR1*. In contrast, the role of *CX3CR1* and atherosclerosis has been clearly confirmed [33, 34].

*Interleukin-32 (IL32)* is described as a pro-inflammatory cytokine, which is involved in the pathogenesis of many inflammatory diseases. It is known to play a role in rheumatoid arthritis because it can induce TNF α, a major cytokine in Rheumatoid Arthritis. In addition, *IL-32* helps to induce other pro-inflammatory mediators, such as procoagulant, pro-inflammatory and cytokine effects of *IL-1* β when siRNA reduces *IL-32* levels, such as IL-1 β-induced ICAM-1 production, which also significantly reduces the up-regulation of ICAM-1 in human umbilical cord endothelial cells (HUVECs) induced by *IL-1 β*, so it is considered that *IL32* plays an important role in the process of atherosclerosis [35, 36]. At the same time, *IL-32* is also highly expressed in T cells and is known to play an important role in the late stage of atherosclerosis, characterized by plaque instability and rupture. In view of these facts, *IL-32* is an important factor promoting the development of CVD in individuals with chronic inflammatory diseases.

*TLR5* is the extracellular receptor of bacterial flagellin and is widely expressed in almost all tissue types.In addition to one or more exogenous stimuli, most tlr also respond to specific endogenous ligands [37]. Although most of the endogenous ligands of *TLRs9* have been described, there is a lack of equivalent ligands for *TLR5*. Since many exogenous TLR ligands are expressed in atherosclerotic lesions, flagellin may also play a role in the development of atherosclerosis.Related studies show that *TLR5* deficiency can reduce the formation of atherosclerosis in LDLr-/-mice [38]. In addition, the plaques of these mice contained fewer macrophages and smaller necrotic cores than mice that received WT bone marrow. These results are also expressed, that is, the role of *TLR5* in atherosclerotic plaque formation and inflammatory cell accumulation [39, 40].

Atherosclerosis is increasingly regarded as an inflammatory disease because the inflammatory process plays an important role in all stages of plaque development. It is also considered as a possible mechanism for the adverse consequences of MS [41]. In fact, the level of inflammation in patients with MS may help identify patients who are at high risk of adverse consequences. Inflammation can increase OS by oxidative modification of LDL [42]. The immune response to these modified lipoproteins drives the pathogenicity of plaques by releasing pro-inflammatory mediators, leading to chronic inflammation. Oxidized LDL atherosclerotic products induce the formation of foam cells and fat stripes in the vascular wall, which is a sign of the beginning of atherosclerosis [43]. Therefore, in our study, we analyzed the immune infiltration of atherosclerosis and metabolic syndrome at the same time, and analyzed the correlation of immune infiltration of the screened hub gene.

However, our study still has some limitations, first of all, the data are derived from the GEO public database, rather than RNA-seq through patient specimens, there are some information differences. Secondly, this study is a single-center study, in the clinical verification stage of the sample, the sample size is limited, our results only select the more prominent three genes in the clinical samples for verification.In addition, further animal and cellular studies are needed to confirm the function and mechanism of these genes.

## Conclusion

In this study, we have established a co-expression network between atherosclerotic progression and metabolic syndrome, and identified key genes between the two diseases. Through the method of bioinformatics, we finally obtained 10 hub genes in atherosclerosis and metabolic syndrome, and selected 3 of the most significant genes (*CX3CR1*,* IL32*,* TLR5*) for blood PCR verification. This may be helpful to provide new research ideas for the diagnosis and treatment of AS complicated with MS.

## Data Availability

The datasets analysed during the current study are available in the GEO database: https://www.ncbi.nlm.nih.gov/geo/query/acc.cgi? acc=GSE28829 and https://www.ncbi.nlm.nih.gov/geo/query/acc.cgi? acc=GSE98895.

## References

[1] Song P, Fang Z, Wang H, et al. Global and regional prevalence, burden, and risk factors for carotid atherosclerosis: a systematic review, meta-analysis, and modelling study [J]. Lancet Glob Health. 2020;8(5):e721–9. 32353319 10.1016/S2214-109X(20)30117-0

[2] Zhang S, Liu Y, Cao Y, et al. Targeting the Microenvironment of Vulnerable atherosclerotic plaques: an emerging diagnosis and therapy strategy for atherosclerosis [J]. Adv Mater. 2022;34(29):e2110660. 35238081 10.1002/adma.202110660

[3] Kovanen PT. Mast cells as potential accelerators of human atherosclerosis-from early to late lesions [J]. Int J Mol Sci, 2019, 20(18). 10.3390/ijms20184479 PMC6770933 31514285

[4] Tomaniak M, Katagiri Y, Modolo R, et al. Vulnerable plaques and patients: state-of-the-art [J]. Eur Heart J. 2020;41(31):2997–3004. 32402086 10.1093/eurheartj/ehaa227 PMC8453282

[5] Silveira Rossi JL, Barbalho SM, Reverete de Araujo R, et al. Metabolic syndrome and cardiovascular diseases: going beyond traditional risk factors [J]. Diabetes Metab Res Rev. 2022;38(3):e3502. 34614543 10.1002/dmrr.3502

[6] Parsanathan R, Jain SK. Novel invasive and noninvasive cardiac-specific biomarkers in obesity and Cardiovascular diseases [J]. Metab Syndr Relat Disord. 2020;18(1):10–30. 31618136 10.1089/met.2019.0073 PMC7041332

[7] Fu Y, Xu L, Zhang H, et al. Identification and validation of Immune-related genes diagnostic for progression of atherosclerosis and diabetes [J]. J Inflamm Res. 2023;16:505–21. 36798871 10.2147/JIR.S393788 PMC9926990

[8] Reimers M, Carey VJ. Bioconductor: an open source framework for bioinformatics and computational biology [J]. Methods Enzymol. 2006;411:119–34. 16939789 10.1016/S0076-6879(06)11008-3

[9] Expansion of the Gene Ontology knowledgebase and resources [J]. Nucleic Acids Res. 2017;45(D1):D331–8. 27899567 10.1093/nar/gkw1108 PMC5210579

[10] Kanehisa M, Goto S. KEGG: kyoto encyclopedia of genes and genomes [J]. Nucleic Acids Res. 2000;28(1):27–30. 10592173 10.1093/nar/28.1.27 PMC102409

[11] Vinayagam A, Gibson TE, Lee HJ, et al. Controllability analysis of the directed human protein interaction network identifies disease genes and drug targets [J]. Proc Natl Acad Sci U S A. 2016;113(18):4976–81. 27091990 10.1073/pnas.1603992113 PMC4983807

[12] Vella D, Marini S, Vitali F, et al. MTGO: PPI Network Analysis Via Topological and Functional Module Identification [J]. Sci Rep. 2018;8(1):5499. 29615773 10.1038/s41598-018-23672-0 PMC5882952

[13] Zeng D, Ye Z, Shen R, et al. IOBR: Multi-omics Immuno-Oncology Biological Research to Decode Tumor Microenvironment and signatures [J]. Front Immunol. 2021;12:687975. 34276676 10.3389/fimmu.2021.687975 PMC8283787

[14] Executive Summary of The Third Report of The National Cholesterol Education Program (NCEP). Expert Panel on detection, evaluation, and treatment of high blood cholesterol in adults (Adult Treatment Panel III) [J]. JAMA. 2001;285(19):2486–97. 11368702 10.1001/jama.285.19.2486

[15] Libby P, Buring JE, Badimon L, et al. Atherosclerosis [J]. Nat Rev Dis Primers. 2019;5(1):56. 31420554 10.1038/s41572-019-0106-z

[16] Grundy SM. Metabolic syndrome pandemic [J]. Arterioscler Thromb Vasc Biol. 2008;28(4):629–36. 18174459 10.1161/ATVBAHA.107.151092

[17] Grundy SM, Brewer HB Jr., Cleeman JI et al. Definition of metabolic syndrome: Report of the National Heart, Lung, and Blood Institute/American Heart Association conference on scientific issues related to definition [J]. Circulation, 2004, 109(3): 433-8. 10.1161/01.CIR.0000111245.75752.C6 14744958

[18] Kahn SE, Hull RL, Utzschneider KM. Mechanisms linking obesity to insulin resistance and type 2 diabetes [J]. Nature. 2006;444(7121):840–6. 17167471 10.1038/nature05482

[19] Stamler J, Vaccaro O, Neaton JD, et al. Diabetes, other risk factors, and 12-yr cardiovascular mortality for men screened in the multiple risk factor intervention trial [J]. Diabetes Care. 1993;16(2):434–44. 8432214 10.2337/diacare.16.2.434

[20] Steiner G. Dyslipoproteinemias in diabetes [J]. Clin Invest Med. 1995;18(4):282–7. 8549014

[21] Mulè G, Calcaterra I, Nardi E, et al. Metabolic syndrome in hypertensive patients: an unholy alliance [J]. World J Cardiol. 2014;6(9):890–907. 25276291 10.4330/wjc.v6.i9.890 PMC4176799

[22] Carethers M, Blanchette PL. Pathophysiology of hypertension [J]. Clin Geriatr Med. 1989;5(4):657–74. 2691057 10.1016/S0749-0690(18)30649-9

[23] Formiguera X, Cantón A. Obesity: epidemiology and clinical aspects [J]. Best Pract Res Clin Gastroenterol. 2004;18(6):1125–46. 15561643 10.1016/S1521-6918(04)00091-5

[24] Van Gaal LF, Mertens IL, De Block CE. Mechanisms linking obesity with cardiovascular disease [J]. Nature. 2006;444(7121):875–80. 17167476 10.1038/nature05487

[25] Guh DP, Zhang W, Bansback N, et al. The incidence of co-morbidities related to obesity and overweight: a systematic review and meta-analysis [J]. BMC Public Health. 2009;9:88. 19320986 10.1186/1471-2458-9-88 PMC2667420

[26] Williams CM, Lovegrove JA, Griffin BA. Dietary patterns and cardiovascular disease [J]. Proc Nutr Soc. 2013;72(4):407–11. 23953031 10.1017/S0029665113002048

[27] Yusuf S, Hawken S, Ounpuu S, et al. Obesity and the risk of myocardial infarction in 27,000 participants from 52 countries: a case-control study [J]. Lancet. 2005;366(9497):1640–9. 16271645 10.1016/S0140-6736(05)67663-5

[28] de Koning L, Merchant AT, Pogue J, et al. Waist circumference and waist-to-hip ratio as predictors of cardiovascular events: meta-regression analysis of prospective studies [J]. Eur Heart J. 2007;28(7):850–6. 17403720 10.1093/eurheartj/ehm026

[29] Whitlock G, Lewington S, Sherliker P, et al. Body-mass index and cause-specific mortality in 900 000 adults: collaborative analyses of 57 prospective studies [J]. Lancet. 2009;373(9669):1083–96. 19299006 10.1016/S0140-6736(09)60318-4 PMC2662372

[30] Jr. Genest J J G. Dyslipidemia and coronary artery disease. Can J Cardiol. 2000;16(Suppl A):a3–4. 10653923

[31] Ninomiya JK, L’Italien G, Criqui MH, et al. Association of the metabolic syndrome with history of myocardial infarction and stroke in the Third National Health and Nutrition Examination Survey [J]. Circulation. 2004;109(1):42–6. 14676144 10.1161/01.CIR.0000108926.04022.0C

[32] Imai T, Hieshima K, Haskell C, et al. Identification and molecular characterization of fractalkine receptor CX3CR1, which mediates both leukocyte migration and adhesion [J]. Cell. 1997;91(4):521–30. 9390561 10.1016/S0092-8674(00)80438-9

[33] Combadière C, Potteaux S, Gao JL, et al. Decreased atherosclerotic lesion formation in CX3CR1/apolipoprotein E double knockout mice [J]. Circulation. 2003;107(7):1009–16. 12600915 10.1161/01.CIR.0000057548.68243.42

[34] Lesnik P, Haskell CA, Charo IF. Decreased atherosclerosis in CX3CR1-/- mice reveals a role for fractalkine in atherogenesis [J]. J Clin Invest. 2003;111(3):333–40. 12569158 10.1172/JCI15555 PMC151849

[35] Nold-Petry CA, Nold MF, Zepp JA, et al. IL-32-dependent effects of IL-1beta on endothelial cell functions [J]. Proc Natl Acad Sci U S A. 2009;106(10):3883–8. 19228941 10.1073/pnas.0813334106 PMC2656174

[36] Heinhuis B, Popa CD, van Tits BL, et al. Towards a role of interleukin-32 in atherosclerosis [J]. Cytokine. 2013;64(1):433–40. 23727326 10.1016/j.cyto.2013.05.002

[37] Rifkin IR, Leadbetter EA, Busconi L, et al. Toll-like receptors, endogenous ligands, and systemic autoimmune disease [J]. Immunol Rev. 2005;204:27–42. 15790348 10.1111/j.0105-2896.2005.00239.x

[38] Ellenbroek GH, van Puijvelde GH, Anas AA, et al. Leukocyte TLR5 deficiency inhibits atherosclerosis by reduced macrophage recruitment and defective T-cell responsiveness [J]. Sci Rep. 2017;7:42688. 28202909 10.1038/srep42688 PMC5311952

[39] Zhang Y, Zhang Y. Pterostilbene, a novel natural plant conduct, inhibits high fat-induced atherosclerosis inflammation via NF-κB signaling pathway in toll-like receptor 5 (TLR5) deficient mice [J]. Biomed Pharmacother. 2016;81:345–55. 27261612 10.1016/j.biopha.2016.04.031

[40] Zarember KA, Godowski PJ. Tissue expression of human toll-like receptors and differential regulation of toll-like receptor mRNAs in leukocytes in response to microbes, their products, and cytokines [J]. J Immunol. 2002;168(2):554–61. 11777946 10.4049/jimmunol.168.2.554

[41] van Diepen JA, Berbée JF, Havekes LM, et al. Interactions between inflammation and lipid metabolism: relevance for efficacy of anti-inflammatory drugs in the treatment of atherosclerosis [J]. Atherosclerosis. 2013;228(2):306–15. 23518178 10.1016/j.atherosclerosis.2013.02.028

[42] Steinberg D. The LDL modification hypothesis of atherogenesis: an update [J]. J Lipid Res. 2009;50(SupplSuppl):S376–81. 19011257 10.1194/jlr.R800087-JLR200 PMC2674707

[43] García-González V, Delgado-Coello B, Pérez-Torres A, et al. Reality of a vaccine in the Prevention and Treatment of atherosclerosis [J]. Arch Med Res. 2015;46(5):427–37. 26100340 10.1016/j.arcmed.2015.06.004

